# An Unusual Case of Post-Partum Hemorrhage

**Published:** 1886-11-20

**Authors:** P. C. Williams


### AN UNUSUAL CASE OF POST-PARTUM HÆMORRHAGE.

Reported at Baltimore Gynæcological Society

BY P. C. WILLIAMS, M.D.

Strictly speaking post-partum hæorrhage is limited to the puerperal process attending or immediately succeeding the third stage of labor. In that sense, the case I am about briefly to report is incorrectly named, but it is difficult to designate it in other terms, and I have ventured to call it "An unusual case of post-partum hæmorrhage." February 1, 1886, Mrs. L., a strong, healthy, well-formed woman was confined with her first child. The labor presented no complication, and was completed within a reasonable time under the influence of a moderate quantity of chloroform. The placenta was examined and was found to have come away entire, with but very slight loss of blood. There was an abundant flow of milk on the third day. The convalescence progressed perfectly until the fifth day, when I was sent for with great urgency. I was soon at the house and found the lady flooding violently, the bed filled with blood and the woman pulseless and prostrated to an alarming degree. The nurse had already given two teaspoonful doses of fluid extract of ergot and had applied ice freely to the abdomen. Placing my hand on the abdomen I found it filled with the womb, which was distended to the size of an eighth-month pregnancy. Recognizing the gravity of the position, I immediately administered hypodermically a drachm of fluid extract of ergot. I then inserted my hand and emptied the womb of the clots which had so largely distended it. As soon as it was emptied, I made constant, strong pressure upon the abdomen, and soon found that the ergot began to act and produce decided contractions of the womb. I then gave another hypodermic dose of ergot, which, with the continued pressure upon the abdomen, *maintained* the uterine contraction and the hemorrhage was checked and never returned. The woman was frightfully reduced by the great loss of blood which she had experienced, but she soon began to rally and went on to a steady and complete restoration of health.

I was at great loss to explain the cause of this excessive and unexpected hæmorrhage. I had seen my patient at 10 o'clock that morning, when she was apparently perfectly well. At 1 o'clock that night was sent for and found the condition I have described. What could have produced the hæmorrhage? The woman was about twenty years old, had always enjoyed uninterrupted health, (I had known her since her birth) had had no trouble during her period of pregnancy, her confinement was a little tedious but perfectly natural, there was very slight loss of blood during the labor, the placenta was expelled *entire*, and the progress of the case was unusually satisfactory until the eighth day, when the sudden change took place that produced the formidable hæmorrhage above described. Upon careful inquiry, I

finally ascertained, through the lady's husband, that she was in the habit of putting herself to sleep by the inhalation of *chloroform liniment*, with which she saturated a handkerchief and applied it over her nose and mouth. This liniment consisted of 2 parts tinct. camphor and 1 part each tinc. aconite and chloroform. She had inhaled this liniment night after night for several weeks before her confinement, and had gradually increased the quantity, until she used *eight to ten ounces every night*. The night in question she had used it with unusual freedom, and at 1 o'clock it had affected her so profoundly as to produce this alarming hæmorrhage. She was made to understand the great danger of her continuing the inhalation of the liniment and readily consented to abandon its use entirely. For a few nights I gave hypodermic doses of morphia to secure necessary sleep.

The dose of morphia was gradually diminished, and after *ten days* was wholly discontinued. This case interested me greatly:

1. It was the first of the sort that I had ever seen.

2. It proved the great power of the hypodermic use of ergot in controlling uterine hæmorrhage. In this case, as in others in which I have used it, its effect was almost instantaneous.

3. It is wonderful that any one could habitually inhale a mixture containing so much aconite, viz., 2 ounces to the half-pint, and experience so little constitutional injury, as, both before and after the hæmorrhage referred to, her health has been perfectly good and has so continued until the present time.

Dr. B. B. Browne asked Dr. Williams if any remains of placenta or membranes were found in the clots passed by his patient, and cited a case reported by Dr. Coskery before the Clinical Society. In this case examination of the placenta seemed to show that it came away entire, but on the inner surface of the uterus, which was removed post-mortem, there was quite a mass of placental tissue. Dr. Browne referred to a case of post-partum hæmorrhage, which he had recently seen, in which the fluid extract of ergot, injected hypodermically, seemed to produce rapid contraction of the uterus, but, as an intra uterine injection of equal parts of vinegar and very hot water was used at the same time, it was impossible to tell which was the most active in checking the hæmorrhage.

Dr. C. H. Riley said he had seen one case somewhat smilar to Dr. Williams'. The patient, a primiparæ, got along very well for about a week following labor. At this time, to determine the exact position of the uterus, he introduced a sound with great care, but the examination was followed by profuse flooding. He tamponed the vagina, and left the tampon in two or three days. There was no return of the hæmorrhage.

Dr. G. Lane Taneyhill stated that he had used Bonjeau's preparation of ergotine hypodermically with admirable results in cases of post-partum hæmorrhage. Thirty grains of the ergotine were dissolved in 450 drops of glycerine, and 20 drops of this solution were injected. It was not necessary to repeat the injection.

Dr. T. A. Ashby remarked that whilst the treatment of post-partum hæmorrhage was being considered, he would say that he had had an experience with vinegar as hæmostatic in post-partum hæmorrhage which confirmed his opinion in regard to its great value in cases where hæmorrhage could not be controlled with ergot and other agents. He then related a case of violent hæmorrhage coming on at the time of delivery from an atonic and fagged-out uterus. He gave ergot hypodermically twice, injected hot water into the uterus, used pressure and taxis, still the uterine contraction was unsatisfactory, and the loss of blood was kept up. He next called for vinegar. A half gallon or more was emptied into a basin, and with a Davidson's syringe, a stream was quickly injected into the uterine cavity. Before the basin was emptied the uterus began to contract firmly. Hæmorrhage ceased promptly and did not return. Dr. Ashby believes that vinegar acts both as a hæmostatic and as an antiseptic. He favored the plan of using a syringe instead of a sponge, as recommended by the late Dr. Penrose. The long tube of the syringe could be carried well into the uterine cavity. There was less danger in doing this than from the introduction of the hand.

Dr. George W. Miltenberger said that Penrose and Wallace had stated that they considered vinegar, applied to the inner surface of the uterus, the most powerful hæmostatic in use. They had never known it to fail either in their own hands or in those of their students.

Dr. L. E. Neale related a case from his own practice (hospital) of secondary post-partum uterine hæmorrhage, occuring on the *ninth* puerperal day, and resulting fatally. The patient, an Irish woman, age twenty-five years, primipara, was delivered by low forceps operation, at the University Hospital, March 1885. The cervix and perineum were uninjured, the placenta came away entire, the third stage being normal. A mild attack of puerperal fever readily yielded to appropriate treatment. She was considered out of all danger and in excellent condition, when on the *ninth* puerperal day, in the abscence of all attendants, a violent uterine hæmorrhage occurred and ceased spontaneously, leaving her moribund. She died on the following morning. Dr. N. considered the hæmorrhage in this case too profuse and sudden to be explained otherwise than by some form of atony of the uterus. He had never attended a case of severe or dangerous post-partum hæmorrhage, "*flooding*," in his own private practice, but, from what he had been taught and had clinically observed, he thought the immediate introduction of the hand in utero, (the obstetrician's hand whilst attending a case of labor should always be aseptic) with or without ice and squeezing the uterus between the hand without and the *first* within, the quickest and surest means of relief. He would also use ergot hypodermically.

Dr. Williams remarked that some years ago he reported before the Medical and Chirurgical Faculty of Maryland some cases in which he had used ergot hypodermically in post-partum hæmorrhage. In one case, he first passed ice within the uterine cavity, then his hand, and scratched the lining membrane, without producing any contractions. He then injected ergot into the thigh and as soon as possible reintroduced his hand into the uterus, when, almost immediately it contracted firmly. This was the case which suggested to him the hypodermic use of ergot. He considered the fluid extract of ergot a more reliable preparation than ergotine. He makes it a rule to instruct every woman, whom he is engaged to attend in labor, to have on hand chloroform and fluid extract of ergot and he always gives ergot at the end of the labor.

Dr. H. P. C. Wilson said he always followed the rule laid down by Dr. Williams in requesting his patients to have chloroform and fluid extract of ergot on hand before labor begins, but never gives ergot before the expulsion of the child, and only then when there is any indication for its use. We have, in the hand introduced into the uterus, the means of promptly arresting post-partum hæmorrhage, while other agents, to be used if necessary, have time to secure permanent contraction of the organ. He had confidence in the *hand* as a *curette*, in hot water and in ergot, in the above cases, but he did not approve of giving ergot after every case of labor, as it insured to the large majority of women, unnecessary suffering in excessive after pains, and he only used it in cases where there were indications of the occurrence of excessive hæmorrhage. He could recall one case where ergot by the mouth, rectum and hypodermically failed to control the hæmorrhage and when manipulation of the uterine cavity with the fingers was equally inefficient. The uterus would contract and expand again and again under these remedies, and the hemorrhage, with each expansion, was frightful. This woman was saved, when almost moribund, by passing the hand into the uterus and, with long finger nails, raking the whole mucous surface *thoroughly* for several minutes. She lost no more blood after this manipulation.

In another case of post-partum hæmorrhage, when ergot, and the hand and ice in the uterine cavity failed, he had saved the woman, when cold and pulseless, by throwing very hot water into the uterus. In this case a pint or two of hot water would cause the uterus to contract and check the bleeding, but so soon as irrigation was stopped, the uterus would expand, and it was only after pulling the woman's hips over the edge of the bed, with a tub under her, and pumping in gallons of hot water, that he succeeded in producing permanent uterine contraction, and arresting the hæmorrhage. With the means now at command, Dr. W. had come to the conclusion, that very few, if any should die of post-partum hæmorrhage.

Dr. W. E. Mosely had found intra-uterine injections of hot water a very certain method of checking hæmorrhage from the endometrium. It must be used in large quantities and hot, not less than 115° or 120° F.—*Maryland Medical Journal.*

**M. Casanow** (Russia) states that tannate of mercury renders syphilis innocuous within one year; that in that time a syphilitic person may marry with impunity, provided that he continues the use of iodide of sodium for the next year, and provided that no syphilides, periostitis, or ulcerations remain. Relapse also is very uncommon, he says, under this treatment.—*Ibid.*

